# Factors influencing environmental sampling recovery of healthcare pathogens from non-porous surfaces with cellulose sponges

**DOI:** 10.1371/journal.pone.0261588

**Published:** 2022-01-13

**Authors:** Laura J. Rose, Hollis Houston, Marla Martinez-Smith, Amanda K. Lyons, Carrie Whitworth, Sujan C. Reddy, Judith Noble-Wang

**Affiliations:** Division of Healthcare Quality Promotion, National Center for Emerging and Infectious Diseases, Centers for Disease Control and Prevention, Atlanta, Georgia, United States of America; Public Health England, UNITED KINGDOM

## Abstract

Results from sampling healthcare surfaces for pathogens are difficult to interpret without understanding the factors that influence pathogen detection. We investigated the recovery of four healthcare-associated pathogens from three common surface materials, and how a body fluid simulant (artificial test soil, ATS), deposition method, and contamination levels influence the percent of organisms recovered (%R). Known quantities of carbapenemase-producing KPC+ *Klebsiella pneumoniae* (KPC), *Acinetobacter baumannii*, vancomycin-resistant *Enterococcus faecalis*, and *Clostridioides difficile* spores (CD) were suspended in Butterfield’s buffer or ATS, deposited on 323cm^2^ steel, plastic, and laminate surfaces, allowed to dry 1h, then sampled with a cellulose sponge wipe. Bacteria were eluted, cultured, CFU counted and %R determined relative to the inoculum. The %R varied by organism, from <1% (KPC) to almost 60% (CD) and was more dependent upon the organism’s characteristics and presence of ATS than on surface type. KPC persistence as determined by culture also declined by >1 log_10_ within the 60 min drying time. For all organisms, the %R was significantly greater if suspended in ATS than if suspended in Butterfield’s buffer (p<0.05), and for most organisms the %R was not significantly different when sampled from any of the three surfaces. Organisms deposited in multiple droplets were recovered at equal or higher %R than if spread evenly on the surface. This work assists in interpreting data collected while investigating a healthcare infection outbreak or while conducting infection intervention studies.

## Introduction

Contaminated healthcare surfaces such as bedrails, overbed tables and shared medical equipment can contribute to pathogen transmission and healthcare-associated infections (HAI) [1–4]. Many organisms associated with HAIs are known to persist for extended periods on fomites [5–8] and organism transfer indirectly and directly from patients to surfaces has been demonstrated [9, 10].

Although environmental contamination can lead to transmission events, studies have not been able to show that any particular level of microbial contamination is associated with increased risk of acquiring a HAI [11]. For this reason, routine monitoring in healthcare facilities has not been required or recommended [12–14]. While standard methods exist for sampling food preparation surfaces [15, 16] and pharmaceutical manufacturing surfaces [17, 18] no standard methods or practices exist for sampling healthcare surfaces [19]. Meanwhile, targeted sampling is frequently done for epidemiological investigations [1, 20–22], disinfection efficacy evaluations [1, 23–25], evaluation of antimicrobial surfaces [26, 27], studies evaluating pathogen transfer to hands, gloves, and gowns [28, 29], and investigations of the role of laundry practices in HAI [30, 31]. Understanding the factors that influence the collection and culturing of organisms from surfaces, and the limitations of the methods is critical for correctly interpreting the results of the sampling event. Most environmental sampling studies conducted in a clinical setting use swabs (cotton, foam or flocked tipped) or contact plates on small surface areas (most close to 100 cm^2^) [32–34] and report either the presence/absence of a target organism, or the percent of surfaces positive [35, 36]. These types of studies do not articulate the limitations of the methods or the implications of these limitations on interpreting results. When studies are conducted in a clinical setting, the original bioburden present on a surface is not known, and therefore, regardless of whether all other factors are uniform, it is impossible to determine the efficiency and therefore the sensitivity of the sampling method.

Previous work has demonstrated that the recovery efficiency (percent of the actual, known quantity of bioburden or target organism detected from a surface) of a sampling method is dependent on several factors including 1) the material the sampling tool is composed of (cotton, polyester, cellulose, nylon flock, etc.), 2) the premoistening fluid used [37], 3) the surface area sampled [38, 39], 4) the transport conditions [40], and 5) the elution method [41]. In addition, the media selection can influence whether a target organism is detected [42, 43]. The presence of complex matrices such as blood, saliva, mucin or stool may influence the adherence of bacteria to surfaces [44]. The presence of these matrices and other microorganisms may also influence the cell surface characteristics [45, 46], and therefore influence adherence and/or release from healthcare surfaces and sampling materials.

Studies with standard swabs (foam or flocked polyester tipped) have shown that the efficiency decreases as the sampled area increases [38, 39], but investigators understandably prefer to sample the largest area to capture more organisms. Since contamination may not be uniform in the healthcare environment and may occur in isolated ‘hot spots’ (random dispersion of pathogens), sampling larger surface areas may improve detection of the target organism or better characterize the room contamination. Sponge wipes are able to sample larger surface areas than standard swabs and have been shown to be efficient and sensitive in sampling *B*. *anthracis* spores [47, 48]. These sponge wipes have also been used frequently in epidemiological investigations [31, 49], yet to our knowledge, no work has yet been published describing the efficiency of sponge sampling materials for healthcare pathogens (i.e. those of concern in transmission of infections within healthcare facilities).

Many considerations come into play when choosing a sampling tool and designing a sampling plan, such as the surface area to sample, premoistening liquid, transport conditions, and elution methods [19, 50, 51]. The objective of this study was to investigate some of the factors that influence the sampling efficiency of cellulose sponge wipes for recovery of four antimicrobial-resistant bacterial pathogens from environmental surfaces. Five variables were included in these evaluations: 1) presence or absence of organic and inorganic substances; 2) deposition as isolated drops (to simulate ‘hot spots’) as compared to evenly spread inoculum, 3) inoculum level, 4) use of selective vs. non-selective culture media for detection of recovered organisms, and 5) characteristics of the surface materials. To assess the microbial differences that may impact detection of recovered organisms, the persistence of the organisms on the surfaces was also investigated.

## Materials and methods

### Bacterial growth and inoculum preparation

Carbapenemase-producing KPC+ *Klebsiella pneumoniae* BAA-1705 (KPC), multi-drug resistant *Acinetobacter baumannii* MLST type 12 (AB) and vancomycin-resistant *Enterococcus faecalis* A256 (VRE) were grown on Trypticase soy agar with 5% sheep blood (TSA II, BD, Franklin Lakes, NJ) 18–24 h. A 0.5 McFarland standard (10^8^ CFU/mL) was prepared in PBS with 0.02% Tween 80 (PBST). *Clostridioides difficile* spores ATCC 43598 (CD) were prepared as described previously [52], resulting in a working stock suspension of 10^7^ CFU/mL of spores in PBST.

The cells and spores were suspended and adjusted to 10^7^ or 10^8^ CFU/mL in PBS with 0.02% Tween 80 (to disrupt cell aggregates), then diluted in series so that the final inocula suspensions of 10^6^ or 10^4^ CFU/mL were in either Butterfield’s buffer (BB) alone, BB with 20% Artificial Test Soil (ATS, Healthmark Industries Company, Inc., Frasier MI) or 20% ATS with 10 mg/mLdust added (dust; A-3 Medium Test Dust, Powder Technologies, Inc. Burnsville, MN). The ATS was selected as a body fluid simulant, as it contains albumin, hemoglobin, amino acids, and vitamins. The test dust was selected to simulate soil that may be carried inside a healthcare facility in small amounts from outside, via a window, air handling unit, or on visitor’s clothes, and it contains inorganic dust, fungal spores, *Bacillus* spp. spores, actinomycetes and yeast [47].

Cells were characterized for hydrophobicity using the water contact angle method described by Busscher et al. [44]. Briefly, the cells were filtered onto a membrane to create a lawn, dried for 60 min., then the water contact angle was measured using a confocal microscope (LEXT™ OLS4000, Olympus, Miami, FL). The zeta potential of cells, a proxy for cell surface charge, was measured using a ZetaSizer (Malvern Instruments, Westborough, MA). Both tests were conducted on cells suspended in BB and in 20% ATS.

### Preparation of surface materials and inoculation

**Fig 1:**
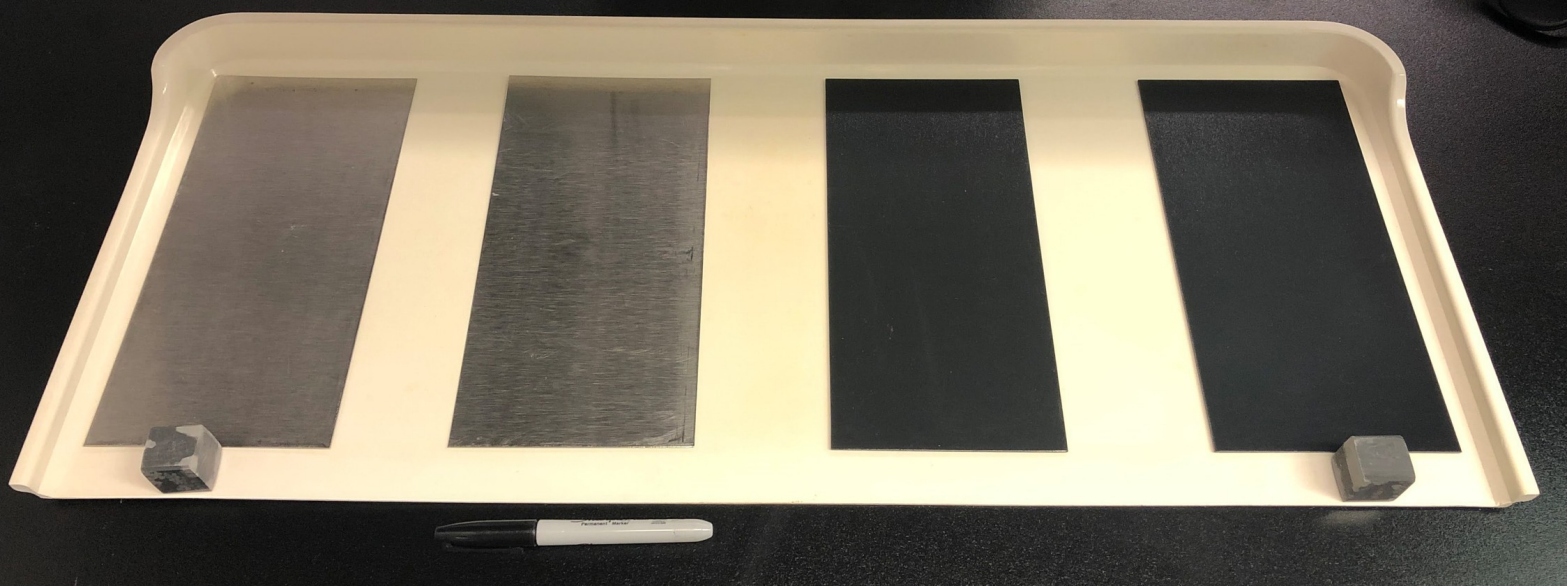
Stainless steel and textured plastic test surface.

**Fig 2:**
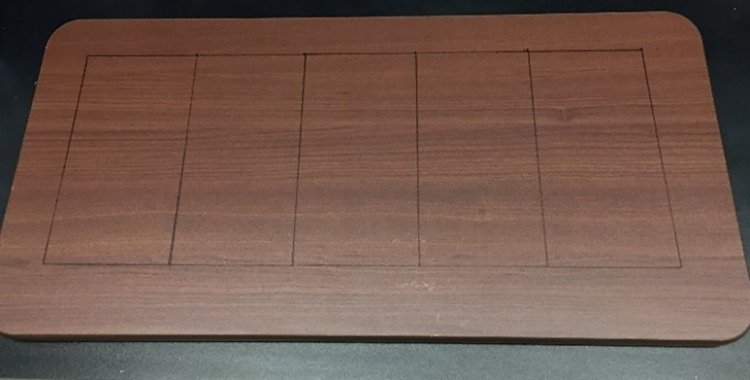
Wood grain laminated hospital tabletop test surface.

Test surfaces were sections (coupons) of stainless steel (SS, T-304 alloy, 24-gauge, Steward Stainless Supply, Inc., Suwanee, GA), textured plastic (TP, Kydex-T, 0–80 thickness, P1 Haircell texture) and laminate (WL, Wood grain laminated hospital tabletop, T-molded edge, Invacare, Inc. UPC# 066510030777) (Figs 1 and 2). For sampling evaluations 322.6 cm^2^ (50 in^2^) coupons were used, and for persistence evaluations 4cm^2^ coupons were used. All surfaces were analyzed for roughness (profilometer, Ramé-Hart Instrument Co. Succasunna, NJ), contact angle (confocal microscope) and zeta potential (ZetaSizer, Malvern Panalytical Inc., Westborough MA) measurements. All surfaces were cleaned prior to use by scrubbing twice with non-antimicrobial soap (Versaclean, Fisher Scientific, Sewanee, GA) rinsing with reverse osmosis water, then spraying with 70% ethanol, allowing for a 1 min contact time, and then wiping with a lint-free towel. Steel coupons were sterilized by autoclaving, while acrylic alloy and laminate coupons were sterilized by UV irradiation for 1 hr.

### Sampling and sample processing

Sterile 322.5 cm^2^ coupons were inoculated with 500 μL of the bacteria suspended in either BB, ATS, or ATS plus dust (resulting in inoculum of 10^4^ or 10^6^ CFU/coupon), then spread uniformly across the separate coupons with a cell spreader. These experiments were conducted to determine the influence of the organic matter, the inoculum level, and the effect of selective culture media. These evaluations were conducted on at least two separate days, with 3 replicates for each parameter being compared, and one negative control coupon each day. Negative controls consisted of inoculating with suspending media only, no organisms.

**Fig 3:**
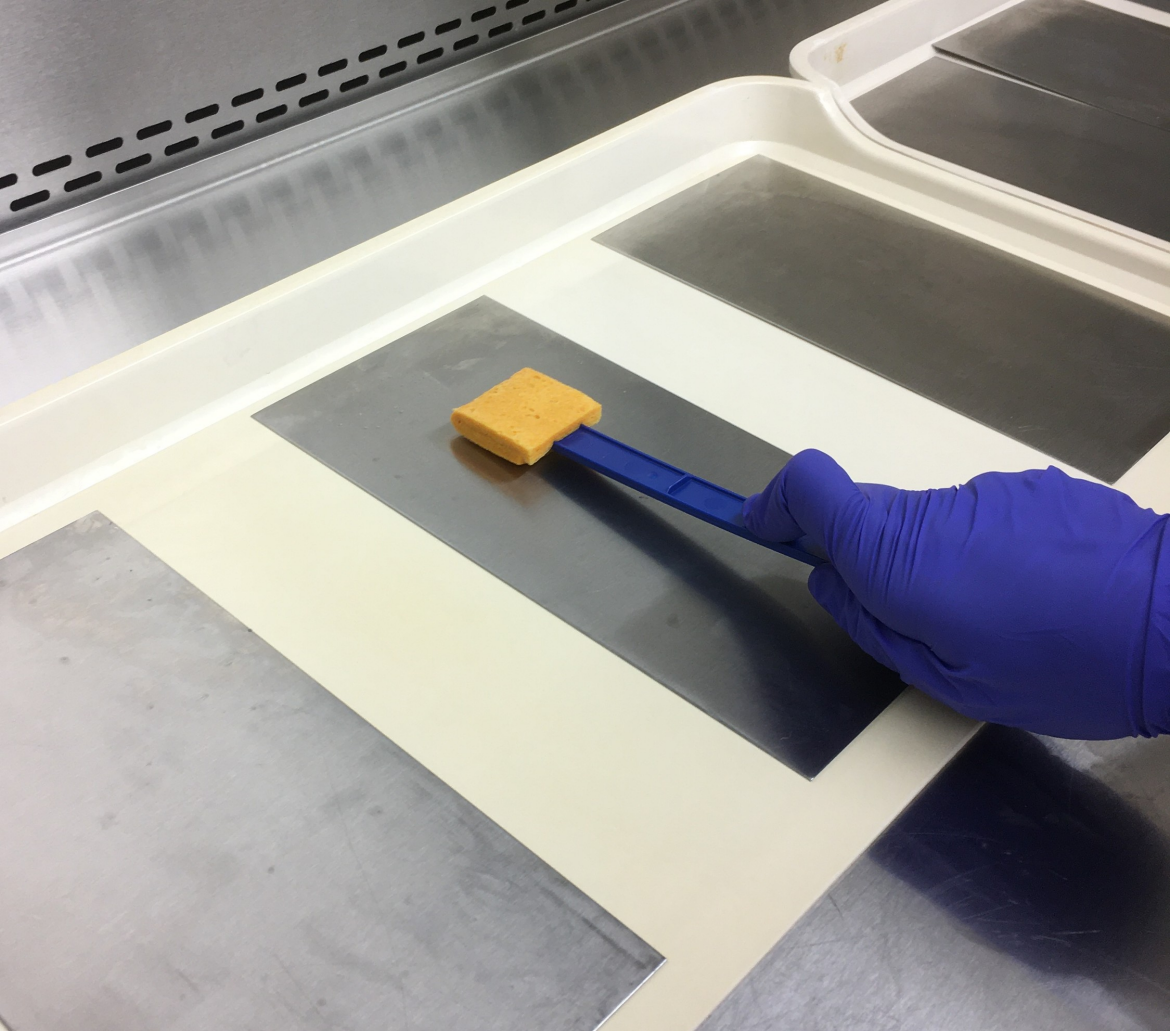
Sponge sampling of steel surface.

**Fig 4:**
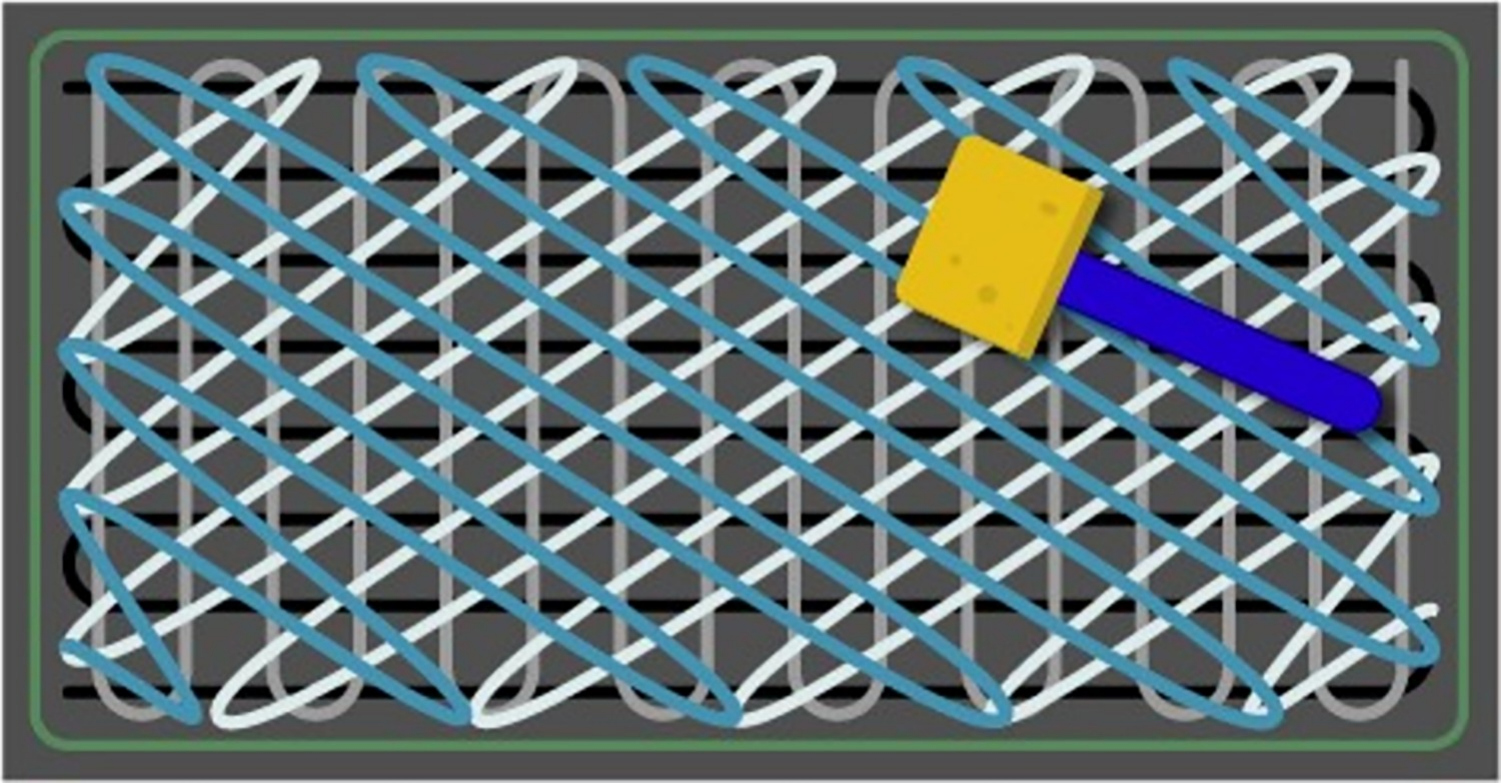
Sponge moved in multiple overlapping directions to cover the entire surface.

Based on the findings of this first set of experiments (improved recovery when ATS present), subsequent evaluations were conducted with cells suspended only in ATS. To compare evenly spread inocula with simulated “hot spots”, cells of VRE and AB were deposited in a 4 cm^2^ area in the center of 322.5 cm^2^ steel and plastic coupons. These evaluations were completed with inocula of 10^6^ and 10^4^ CFU/coupon. After inoculation, coupons were left to dry in a closed biosafety cabinet (air flow off) for 60–90 min (until visibly dry) before sampling began. The sampling of the 322.5 cm^2^ coupon was conducted in the same manner as if the inoculum was spread evenly. Each variable was tested with a minimum of 6 coupons and one negative control coupon for each surface type, inoculation type (hot spot and spread) and organism (VRE and AB). Negative controls consisted of inoculating with suspending media only, no organisms. Sampling of surfaces was performed using cellulose sponges pre-moistened with neutralizing buffer (Sponge-Stick™, P/N SSL10NB; 3M, St. Paul, MN). All sides of the sponges were used to sample the surface in a standardized manner, moving in multiple directions while rotating the sponge, as previously described [47, 53] (Figs 3 and 4). Organisms were eluted from the sponges in 90 mL of PBS.

Organisms were eluted from the sponges in 90 mL of PBS with 0.02% Tween® 80 using a homogenizer (Stomacher® 400 Circulator, Seward Laboratory Systems, Inc.) set at 200 rpm (vegetative cells) or 260 rpm (CD spores) for 1 min. The eluent was divided into two 50 mL conical tubes and concentrated by centrifugation at 2700 rpm for 20 min to pellet and all but ~3 mL of eluent removed from each tube from the tube and discarded. The pellet was then resuspended by vortexing for 2 minutes in 10 second bursts, the resuspended pellets combined into one tube, and the final volume was measured and recorded. This eluent was then diluted in 10-fold series, spread plated, incubated at optimum conditions, and colonies were counted upon incubation (18–24 h, but checked again at 48 and 72h for slow growing colonies). When culturing eluate from samples containing BB or ATS only, non-selective media were used to culture the eluent: Trypticase^TM^ Soy Agar with 5% sheep blood for AB, KPC and VRE (TSA II^TM^ BD BBL, Franklin Lakes, NJ), and Brain Heart Infusion Agar with horse blood and taurocholate for CD (BHI-HT; Anaerobe Systems, Morgan Hill, CA). When culturing eluate from ATS + dust samples, both selective and non-selective media were used to determine whether the media influenced the quantitation of organisms after desiccation. Selective media included MDRA agar (cat #259 Hardy Diagnostics, Santa Maria, CA) for AB, CHROME VRE agar (Hardy Diagnostics) for VRE, MacConkey II agar (Becton Dickson BBL ™, Franklin Lakes, NJ) for KPC, and CCFA-HT (Anaerobe systems, Morgan Hill, CA) for CD. Cultures of AB, KPC and VRE were incubated at 35°C for 24 h, *C*. *difficile* cultures were incubated in an anaerobe chamber at 35^°^C for 48 h.

### Persistence evaluations

In a limited evaluation, the survival of the three vegetative bacteria, KPC, AB and VRE, in both BB and ATS, was investigated over 90 min, the maximum dry time used before sampling. Suspensions of 10^6^ CFU/mL of each organism were made in either BB or ATS, and 10 μL of each suspension was placed onto 4 cm^2^ coupons of stainless steel and textured plastic (n = 3 coupons for each organism and combination of parameters; steel, plastic, ATS, BB). Coupons were allowed to dry at ambient room temperature and humidity (22°C ±2°C, RH range 22–54%) in a closed biosafety cabinet with the airflow off. At 0, 30, 60, 90 min after inoculating, each coupon was placed in a tube containing 5 mL PBST, then vortexed and sonicated in alternating 30 sec intervals, three times each. The eluate was diluted in series and spread plated for culture and enumeration of CFU.

### Statistical analysis

The percent recovery of the sampling evaluations was determined, based on total CFU recovered relative to the known inoculum (CFU) placed on each coupon (total CFU recovered/CFU of inoculum). The log_10_ reduction of cultivable cells for the persistence evaluations were determined relative to the inoculum. Shapiro-Wilk test used to test for normalcy of the data sets, Mann-Whitney test were conducted to discover differences between experimental variables. Statistical significance was delineated if p<0.05.

## Results

### Sampling efficiency

No significant difference in %R was seen between organisms deposited in ATS and ATS + dust on the non-selective media (p<0.05), therefore these two data sets were combined for comparison to the %R of cells deposited in BB. The %R of all organisms sampled from all surfaces was significantly higher when suspended in ATS than when suspended in BB (p<0.01, Table 1). All negative controls were negative for the target organism being tested. The %R was lowest for KPC and highest for CD spores, regardless of surface type or suspending fluid. The mean %R for VRE and AB if suspended in ATS and recovered from all surface types were similar, with no significant difference seen (16.7% and 15.6%, respectively, p = 0.17, Table 1). When suspended in ATS, the mean %R of VRE and AB from plastic was lower than steel and laminate (though never more than a mean difference of 6%), and no significant difference in %R was seen between steel and laminate (p>0.1, Tables 1 and S3). CD spores were detected at a significantly higher %R from plastic than steel or laminate (p = 0.02, but within 6%, Table 1 ATS, S3 Table), and KPC was recovered at a higher %R from steel and plastic than laminate (but within 4%, Table 1 ATS; steel and plastic, p = 0.03, S3 Table).

**Table 1:** Percent recovery (SD) of healthcare-associated pathogens from three surface materials, as suspended in Butterfield’s Buffer (BB) and Artificial Test Soil (ATS), inocula 10^4^ CFU/50in^2^ coupon. All p values ≤ 0.001 when comparing BB and ATS for each given organism and surface type.

Organism	Surface type	BB^1^	ATS^2^
		%R (SD)	%R (SD)
***K*. *pneumoniae* (KPC)**	Steel	0.7 (0.4)	8.9 (3.9)
	Plastic	0.3 (0.3)	7.7 (5.1)
	Laminate	0.6 (0.5)	4.3 (2.0)
	All Surfaces^3^	0.5 (0.5)	6.9 (4.3)
***E*. *faecalis* (VRE)**	Steel	4.1 (1.8)	17.2 (7.3)
	Plastic	3.2 (0.8)	13.2 (5.8)
	Laminate	12.8 (2.8)	19.7 (11.8)
	All Surfaces^4^	6.7 (4.7)	16.7 (9.1)
***A*. *baumannii* (AB)**	Steel	11.7 (2.9)	16.9 (5.9)
	Plastic	6.9 (2.2)	13.7 (5.0)
	Laminate	8.7 (2.7)	16.1 (5.4)
	All Surfaces	9.1 (3.3)	15.6 (5.6)
***C*. *difficile spores (CD)***	Steel	25.1 (9.9)	53.0 (10.3)
	Plastic	32.1 (11.1)	58.9 (12.7)
	Laminate	36.5 (4.7)	52.3 (8.2)
	All Surfaces	31.2 (10.0)	54.7 (10.9)

^1^BB: Cells or spores suspended in Butterfields’ buffer prior to depositing on each surface, n≥ 10.

^2^ATS: Cells or spores suspended in Artificial Test Soil (Healthmark Industries Inc.) prior to depositing on each surface, n≥ 10.

^3^ All Surfaces = mean of %R across Steel, Plastic and Laminate, for the given organism.

### Persistence evaluations

When conducting the sampling evaluations, inocula were dried 60–90 min before sampling. Since ambient conditions in the laboratory varied slightly day to day, the inocula occasionally required more than 60 min to dry before sampling. In the short-term persistence evaluations, we found that KPC and AB declined in cultivability over this time and under these ambient environmental conditions (Table 2). KPC in ATS was found to decline by a mean of 1.08 (SD 0.55) log_10_ CFU in 60 min, while AB declined by only a mean of 0.13 (SD 0.05) log_10_ CFU during the same time period (Table 2). The VRE mean CFU increased slightly after 60 min, a phenomenon known as reductive division, commonly seen in organisms under stress [54, 55].

**Table 2:** Log_10_ change in cultivability (CFU) relative to the inoculum, upon drying 60 or 90 min. Data for steel and plastic pooled. Inocula ranged from 3.9–4.5 log_10_.

Organism	Dry time, min	Log_10_ change in CFU (SD)	Log_10_ change in CFU (SD)	p^3^	n
		BB^1^	ATS^2^		
***K*. *pneumoniae* (KPC)**	60	-0.52 (0.68)	-1.08 (0.55)	0.13	≥9
	90	-2.08 (1.23)	-1.42 (0.31)	0.11	≥9
***A*. *baumannii* (AB)**	60	-0.33 (0.06)	-0.13 (0.05)	0.02	6
	90	-0.29 (0.08)	-0.21 (0.05)	0.17	6
***E*. *faecalis* (VRE) ^4^**	60	-1.50 (0.36)	+0.03 (0.70)	0.2	3
	90	-0.92 (0.40)	+0.08 (0.03)	0.1	3

Note: Inoculum CFU and recovered CFU at each time point were log_10_ normalized, log_10_ reduction determined relative to T0, data from steel and plastic were pooled (for KPC and AB), mean and standard deviations of the log_10_ reductions were determined. Mean log_10_ Inocula: KPC 3.9, AB 4.1, VRE 4.5 log_10_.

^1^BB: Cells or spores suspended in Butterfields’ buffer prior to depositing on each surface.

^2^ATS: Cells or spores suspended in Artificial Test Soil (Healthmark Industries Inc.) prior to depositing on each surface.

^3^ p = Mann-Whitney comparison of log_10_ reduction in CFU over time, as suspended in Buffer as compared to suspended in ATS.

^4^ VRE persistence determined for steel only.

### Inoculum distribution evaluations

Because of the significant decline in cultivable KPC cells, only VRE and AB were evaluated to determine whether the pattern of distribution over the sampling area influenced the %R. We found that sampling of dried droplets or “hot spots” resulted in a greater or equal %R as compared to an evenly distributed dried inoculum (Table 3). A greater %R was seen when inocula were deposited as “hot spots” for AB and VRE on steel, and for VRE on plastic than if evenly distributed. No significant difference was seen between the two methods of inoculation for AB on plastic.

**Table 3:** Percent recovery (SD), Hot Spot vs Even distribution of inoculum (10^4^ CFU/coupon, n = 6 for each variable).

Organism	Surface	Inoculum	% Recovery (SD)	P^1^
***E*. *faecalis* (VRE)**	Steel	Hot Spot	54.1 (9.3)	<0.001
		Even Distribution	21.8 (4.6)	
	Plastic	Hot Spot	34.7 (4.0)	<0.001
		Even Distribution	19.4 (3.3)	
***A. baumannii* (AB)**	Steel	Hot Spot	13.7 (5.2)	<0.001
		Even Distribution	8.2 (2.3)	
	Plastic	Hot Spot	20.2 (9.7)	0.501
		Even Distribution	21.5 (9.9)	

^1^ p = Mann-Whitney comparison of %R of hot spot deposition and even distribution of inoculum prior to drying and sampling. Cells suspended in ATS.

### Inoculum level evaluations

When VRE and AB were placed on the plastic surfaces at two inoculum levels (10^4^ or 10^6^ CFU per 50 in^2^) and spread evenly, no significant difference in % R was seen between the two inoculum levels, with the exception of VRE on steel (Table 4). If spread evenly on steel, the mean % R of VRE was higher at 10^4^/coupon than 10^6^/coupon (25.6% and 20.7%, respectively, p<0.001). If the organisms were placed as a hot spot in the center of the coupon before drying and sampling, no significant difference in %R was seen between the two inoculum levels on either surface type for either organism (Table 4).

**Table 4:** Percent recovery (SD), Inoculum (10^4^ vs 10^6^ CFU/coupon) (n = 6).

			% Recovery (SD)		
Organism	Surface	Inoculum	10^4^	10^6^	p^1^
***E*. *faecalis* (VRE)**	Steel	Hot Spot	62.8 (9.7)	59.7 (6.2)	0.15
		Even Distribution	25.6 (3.7)	20.7 (3.2)	<0.001
	Plastic	Hot Spot	40.3 (13.6)	43.2 (7.0)	0.61
		Even Distribution	14.2 (3.2)	15.4 (4.4)	0.72
		Hot Spot	13.5 (3.8)	13.0 (5.8)	0.46
***A*. *baumannii* (AB)**	Steel	Even Distribution	11.8 (5.4)	9.1 (3.7)	0.13
		Hot Spot	17.2 (3.9)	19.4 (3.7)	0.14
	Plastic	Even Distribution	16.6 (6.6)	16.2 (2.7	0.94

^1^ p = Mann-Whitney comparison of %R of cells deposited at 10^4^ and 10^6^ CFU/coupon.

### Selective media evaluations

Differences in %R when using selective or non-selective media were seen when sampling various surface types. In three out of the twelve parameters (considering the four organisms and three surface types), the mean for the selective media was significantly lower than the non-selective media (Table 5), and in once instance the selective media provided significantly greater %R than non-selective media (CD from plastic; 59.8 and 51.2 respectively, p = 0.001). When data from all surfaces were pooled for each organism, a significant difference was seen in %R using the two media types for KPC and VRE, but not for AB or CD (Table 5). Though some significant differences in %R were observed between selective media and non-selective media, none were greater than 5.6% (AB from steel; Table 5).

**Table 5:** Percent recovery (SD) of four organisms from three surfaces using both selective (indicated by asterisk) and non-selective culture media^1^.

Organism	Surface	Culture medium^2^	%R (SD)	P^3^
***K*. *pneumoniae* (KPC)**	Steel	TSA w/5% SB	9.9 (4.1)	0.86
		MacConkey*	9.5 (2.7)	
	Plastic	TSA w/5% SB	9.9 (5.0)	0.02
		MacConkey*	7.4 (3.8)	
	Laminate	TSA w/5% SB	4.9 (2.0)	<0.001
		MacConkey*	2.6 (1.6)	
	All surfaces pooled	TSA w/5% SB	8.2 (4.5)	= 0.001
		MacConkey*	5.9 (4.1)	
***E*. *faecalis* (VRE)**	Steel	TSA w/5% SB	17.3 (7.8)	0.40
		Chrome VRE*	15.2 (6.0)	
	Plastic	TSA w/5% SB	12.2 (6.7)	0.29
		Chrome VRE*	10.4 (4.6)	
	Laminate	TSA w/5% SB	17.1 (3.8)	<0.001
		Chrome VRE*	10.3 (2.9)	
	All surfaces pooled	TSA w/5% SB	15.5 (6.7)	<0.001
		Chrome VRE*	12.0 (5.2)	
***A*. *baumannii* (AB)**	Steel	TSA w/5% SB	14.5 (5.5)	<0.001
		MDRA*	8.9 (2.4)	
	Plastic	TSA w/5% SB	14.5 (5.6)	0.34
		MDRA*	13.6 (4.0)	
	Laminate	TSA w/5% SB	17.9 (4.9)	0.94
		MDRA*	18.0 (4.5)	
	All surfaces pooled	TSA w/5% SB	15.6 (5.5)	0.003
		MDRA*	13.5 (5.3)	
***C*. *difficile* spores (CD)**	Steel	BHI-HT	53.0 (9.1)	0.07
		CCFA-HT*	49.2 (7.0)	
	Plastic	BHI-HT	51.2 (11.2)	0.001
		CCFA-HT*	59.8 (8.1)	
	Laminate	TSA w/5% SB	50.4 (6.9)	0.19
		CCFA-HT*	48.7 (8.8)	
	All surfaces pooled	BHI-HT	51.9 (8.0)	0.98
		CCFA-HT*	52.5 (9.5)	

^1^ Organisms suspended in ATS+dust, then deposited on each surface and the suspensions dried prior to sampling.

^2^ TSA w/5% SB = Trypticase Soy Agar with 5% sheep blood, MacConkey II from Beckton Dickson BBL^TM^, Chrome VRE from Hardy Diagnostics, MDRA = Multidrug resistant Acinetobacter medium from Hardy Diagnostics, BHI-HT = Brain heart infusion with Horse Blood and Taurocholate from Anaerobe Systems Inc., CCFA-HT = Cycloserine Cefoxitin Fructose Agar with Horse Blood and Taurocholate from Anaerobe Systems Inc.

^3^p = Mann-Whitney comparison of %R of cells or spores cultured on selective or non-selective media.

### Surface roughness, contact angle and zeta potential

The healthcare surfaces used were found to vary in roughness, contact angle and zeta potential (S1 Table). Steel was found to be the smoothest, demonstrate the greatest contact angle (most hydrophobic), and lowest negative charge (-22mV). The laminate was the roughest and had the lowest contact angle (the least hydrophobic) of the three materials tested, and the more negatively charged (-28.4 mV).

### Cell hydrophobicity and zeta potential

The zeta potential measurements of the organisms show CD spores to be the least charged (-4.6 mV in BB, -12.8 mV in ATS) and VRE to be the most charged cells, regardless of suspending fluid (-25.9 mV in BB, -19.6mv in ATS, S2 Table). When cells were suspended in BB and placed on a filter for contact angle measurements, VRE cells were found to be the most hydrophilic (lowest contact angle, 43.5^o^) of the four organisms tested, and CD spores are the most hydrophobic (highest contact angle, 79.6 ^o^) when tested as suspended in BB (S2 Table). When cells were suspended in ATS and placed on a filter for contact angle measurements, VRE was still the most hydrophilic (51.3 ^o^) but interestingly, KPC was the most hydrophobic (85.1 ^o^) of the four organisms tested. When suspended in ATS, the contact angle increased for all vegetative cells as compared to when suspended in BB, while the contact angle for CD spores declined slightly. In contrast, when in ATS, the zeta potential decreased for the vegetative cells and increased for the CD spores.

## Discussion

Surfaces in healthcare settings play an important role in transmission of pathogens [1–4, 56], particularly if cleaning and disinfection is not done properly. The U.S. Centers for Disease Control and Prevention has provided guidance on how to reduce the risk of transmission of HAIs from surfaces, highlighting core components of an environmental disinfection program to ensure appropriate environmental cleaning and disinfection of healthcare surfaces [57]. Effective and efficient surface sampling is a key component to evaluating transmission intervention strategies. Questions often arise as to how to interpret the results of a sampling event. One common question is whether the target organism is truly absent when it is not detected or whether the sampling device is not picking it up. The controlled laboratory studies presented here demonstrate that sampling efficiency varies from one target organism to another, and the presence or absence of an organic matrix surrounding the cells was found to influence the recovery. The % R was always significantly greater when cells were suspended in ATS than when cells were suspended in BB before depositing on surfaces. This improved %R may be attributed to; 1) the organic matrix providing protection from desiccation as seen in the improved persistence as determined by culture for some organisms and time points evaluated, and/or 2) the organic matrix altering the surface properties of the cells or fomite material properties so that cell adherence is reduced as shown by the altered zeta potentials and/or contact angle measurements of the cells and fomite material. The work informs other researchers looking to evaluate sampling tools and processing methods that including an organic body fluid simulant relevant to their study (i.e. blood, saliva, stool) is essential for an accurate assessment of the recovery efficiency.

All the organisms tested here have been previously reported to persist on healthcare surfaces for extended periods of time [5, 8, 56, 58–61]. While this KPC strain was seen to decline by 2 log_10_ in 2 days at 18°C and 20% RH, and 4 log_10_ in 2 days at 26°C and 57% RH, the same AB strain demonstrated <0.5 log_10_ decline in 2 days under the same two controlled conditions [62]. No data for shorter time points under controlled conditions is available. The current work found that culturable cells of the KPC strain declined rapidly by a mean of >1 log_10_ CFU within 60 min (Table 2). Though the current work was conducted at ambient temperature and humidity that varied slightly from day to day, the levels were consistent with those within a healthcare setting with central heating and air conditioning. The variable temperature and RH can influence the cultivability and therefore the %R determination. With KPC in particular, the sponge may be collecting similar numbers of KPC cells as AB or VRE cells, but the KPC cells are not viable or viable but non-culturable (VBNC). If the %R of KPC were to be determined relative to the cultivable cells estimated to be present after drying for 1 h, the overall mean recovery for KPC would be significantly greater than reported as calculated relative to the inoculum cultivable in suspension (lower denominator would result in higher %R). In previous work, the three vegetative organisms evaluated were demonstrated to be metabolically active as measured by esterase activity and a solid phase cytometer (ScanRDI, Biomerieux inc), beyond the times they were detectable by culture [61, 62], indicating they may enter a VBNC state [63–65] when dried on surfaces. Though the potential for VBNC organisms to be present exists, little data is available to estimate the risk of disease transmission from these target pathogens in a VBNC state [66, 67]. In addition to entering the VBNC state, KPC cells may also be more adherent to the surfaces than the other organisms tested here, as the KPC cells were seen to be more hydrophilic, as discussed below.

In addition to protecting the cell and enhancing survival, the presence of organic material can influence the properties of the cell and therefore adherence to surfaces. Many researchers have investigated the cell properties that influence attachment to surfaces, as they relate to biofilm formation [44, 68–70]. The cell properties that can influence adherence include cell surface hydrophobicity and cell surface charge [44, 71] presence of flagella and fimbrae [72] and the production of extracellular polysaccharides (EPS) [73]. This work explored cell hydrophobicity and charge as potential contributors to cell adherence to the steel, plastic, and laminate.

Bacteria in aqueous suspensions are typically negatively charged, and the charge varies between species and strains. The charge can be influenced by the growth medium, pH and ionic strength of the suspending buffer, bacterial growth stage, and bacterial surface structure [74, 75]. The zeta potential is widely used as a proxy for bacterial cell surface charge. The zeta potential is the net electrical charge contained within the region bounded by the slipping plane, a notional boundary between the outer regions of the cell envelope and the ions that interface with the suspending fluid. We measured the zeta potential of the cells as suspended in BB and as suspended in ATS. Cells with a more negative surface charge have been shown to be more likely to adhere to a surfaces [71]. Our zeta potential measurements show that for KPC, VRE and AB, the presence of ATS caused the zeta potential of the cells to become less negative, and therefore less likely to adhere to a surface. These findings are similar to those of Van Merode et al. [76] for *E*. *faecalis*. Interestingly, the zeta potential of CD spores was more negatively charged when ATS was present, indicating a more complex interaction dynamic with these spores.

Water contact angle is an indicator of the hydrophobicity of a cell or a material, and generally the more hydrophobic a cell, the less adherent it will be [77–80]. Though we did not see a direct correlation between cell contact angle and %R across organisms, the contact angle of all vegetative cells increased significantly when ATS was present (though a slight decline for CD spores), indicating increased cell hydrophobicity and reduced adherence. In surface sampling, these cell-surface interactions (charge and hydrophobicity) may play an initial role in cells adhering to surfaces when the inoculum is deposited on a surface, but changes to the cell and the cell-surface dynamic may occur during the 1 h of desiccation prior to sampling. The force applied during sampling and the presence of surfactants and neutralizers in the premoistening fluid of the sponge may also alter cell properties.

Other researchers have shown that cells tend to adhere more to materials that are more hydrophobic [77], and materials that are less negatively charged [81]. The presence of proteins influencing hydrophobicity, charge, and adherence was demonstrated by Barnes et al [82]. Husmark et al. [83] also showed that the pH and polarity of the suspension can influence adherence by altering both the *Bacillus cereus* spore surface and the substrate surface characteristics. The presence of the ATS may have altered the surface charges and/or hydrophobicity of the steel, plastic and laminate [79] by adsorbing onto the fomite surfaces, making them less favorable for attachment through steric effects, or by hydration of the surface to convert it to a more hydrophilic surface [84]. This illustrates that when investigating sampling tools and methods, simulating cell in body fluids on healthcare surfaces by including organic matter such as ATS is important to obtain relevant recovery efficiency data.

The roughness of our three surfaces varied from a mean roughness average (Sa, average of peak and valley of topography) of 0.53 to 7.99 μm (steel, laminate respectively), but the %R for each organism was not influenced by the type of substrate, with the differences in mean %R never exceeding 6.6% for a given organism (if we consider the cells suspended in ATS). Indeed, for VRE, AB, and CD, there was no significant difference between %R from the smoothest (steel) and the roughest (laminate We therefore suggest that the characteristics of the laminate, steel and plastic played a minor part in the sampling efficiencies for these healthcare associated organisms. Instead, we propose that the organism characteristics play the dominant role in %R. Barnes [82] and Hilbert [85] demonstrated that surface roughness had little effect on adherence of bacteria (not identified, rinsed from poultry, counted under SEM), though Arnold et al. [86] reported that surface roughness did influence adherence, indicating that the effect may be organism dependent. Krauter et al. [48] found differences in recovery and false negative rates (FNR) when using the same sponge sampling tool and processing method as used in this study to recover *B*. *anthracis* spores from various surfaces, though the steel and plastic materials were not the same as in the currently reported study; 48.1%R and 9.8%R for roughness averages of 0.13 (steel) and 5.88 μm (plastic panel), respectively. Neither contact angle or zeta potential was reported in the Krauter et al study.

We found that within the range of contamination tested on the surfaces (10^4^ and 10^6^ CFU/50 in^2^), inoculum level did not influence the efficiency of the sampling tool. The “hot spot” inoculation method most likely is a better simulation of how healthcare surfaces are contaminated, and our findings show that recovery in this type of simulation is equal or better than if evenly spread. The improved recovery may be due to improved survival in a denser population of cells and organic matter, or because a smaller proportion of the cells are in direct contact with the fomite surface, and therefore not affected by the forces that influence adherence.

The inoculum level of 10^4^ CFU/50 in^2^ was chosen as one relevant to healthcare surfaces and one that would enable for comparison of the variables. One study [87] reported that the *C*. *difficile* level in the stool of infected patients ranges from 10^4^ to 10^6^ CFU/g stool. Another study found patient colonization of VRE in stool as high as 7.8 (SD 1.5) log_10_ /g and when levels were >4 log_10_/g, environmental samples were positive [88]. Among 402 MRSA colonized patients, O’Hara et al. [89] found the median level of colonization found with nares swabs was 445 CFU/mL of swab eluate. Shams et al. [90] found the burden of some pathogens to be as high as 13,000 CFU/100 cm^2^ (MRSA) and 1680 CFU/100 cm^2^ (VRE) on some hospital surfaces sampled. We have shown that the %R is similar at two inocula levels; 10^4^ to 10^6^ log_10_ CFU/50 in^2^. More extensive work was conducted, using this same sampling tool and processing method at much lower inoculum levels of *B*. *anthracis* spores, and the % R was found to be linear relative to inoculum levels [48].

We cultured our eluate on selective and non-selective media to determine if organisms stressed by desiccation would be further stressed by the selective media that might be used when sampling in a healthcare environment where background organisms would need to be suppressed. Though some significant differences in %R were observed between the selective media and non-selective media, when recoveries from all surface types were pooled for each organism, the means were within 3% of each other (Table 4).

We are aware that this laboratory simulation of sampling investigated only one isolate of each organism, and that variations may be seen between isolates. The ATS was chosen as a suspension matrix because of its formulation to simulate a general body fluid (hemoglobin, albumin, amino acids and vitamins) but suspension in other matrices such as whole blood, urine, mucin and stool also warrant investigation as to their influence on recovery results. If the cell suspensions were dried longer than 60–90 min, or if cells were deposited as a simulated skin shedding, the %R may be different than reported here. Additionally, the cellulose sponge material may differ in its ability to recover and release these organisms into the elution liquid when compared to another sampling material [91], and differences in media selection and/or formulations may influence %R.

## Conclusion

When sampling for healthcare pathogens on surfaces, whether one needs to know the quantity of pathogens present or only if present at all, understanding the efficiency of the sampling method is essential for confidence in the findings. We confirm that the organic matrix contributes to cell survival as determined by culture and influences the adherence of the cell to the surface. Additionally, even though the roughness, contact angle and zeta potential of the three surface types evaluated were different, no significant differences in %R were seen when sampling a given organism from each of the surfaces when the organic matrix was present, indicating that the organism characteristics play a larger role in the sampling efficiency determined here.

When detection is via culture alone, quantitative recovery of the organisms may be underestimated. For example, if the efficiency of a sampling tool is 13% (as for AB from plastic) then we should interpret those results with the understanding that the actual quantity of the bacteria on the surface may be almost one log_10_ higher than what is recovered. Whether the cells are transmissible by touch-transfer from surface to healthcare worker and/or patient remains to be investigated. Additionally, more work is needed to investigate whether these healthcare pathogens enter a VBNC state and if revived, whether they are virulent and a threat to patients.

The data presented here supports the concept that when testing the efficiency of sampling tools to use in field investigations, researchers should simulate the conditions of deposition and include an organic matrix simulating what may be seen on healthcare surfaces. While choice of sampling tool may depend on many factors [50], this work confirms the utility of the cellulose sponge as a sampling tool and contributes to the improvement of environmental sampling strategies for investigation of sources of healthcare contamination. These concepts may help assist with better study design when conducting and interpreting transmission intervention studies.

## Supporting information

S1 Table Surface material roughness, hydrophobicity and zeta potential. (DOCX)

S2 Table Percent recovery (SD) all materials pooled, contact angle and zeta potential of test organisms. (DOCX)

S3 Table Mann-Whitney comparison of percent recovery, as cultured on non-selective media, between surface types for each organism suspended in Artificial Test Soil (ATS) and spread evenly on surfaces. (DOCX)
