# Supplementary material for: Factors influencing environmental sampling recovery of healthcare pathogens from non-porous surfaces with cellulose sponges
Source: PLoS One. 2022 Jan 13;17(1):e0261588. doi: 10.1371/journal.pone.0261588 (PMC8757884; doi:10.1371/journal.pone.0261588)
Supplement: S1 Table — (DOCX) [file pone.0261588.s001.docx]

S1 Table. Surface material Roughness, Hydrophobicity and Zeta Potential.

| **Surface Material** | **Roughness (Sa)^1^** | **Contact Angle^2^ (SD)** | **Zeta Potential (mV)** |
| --- | --- | --- | --- |
| Stainless Steel | 0.53 | 89.47 (6.36) | -22.4 |
| Textured Plastic | 4.29 | 80.97 (3.63) | -23.6 |
| Laminate | 7.99 | 46.31 (5.02) | -28.4 |

^1^Sa- the overall measure of roughness, µm

^2^ mean of 3 measurements
