# Supplementary material for: Factors influencing environmental sampling recovery of healthcare pathogens from non-porous surfaces with cellulose sponges
Source: PLoS One. 2022 Jan 13;17(1):e0261588. doi: 10.1371/journal.pone.0261588 (PMC8757884; doi:10.1371/journal.pone.0261588)
Supplement: S2 Table — (DOCX) [file pone.0261588.s002.docx]

| S2 Table. | Percent Recovery (SD) all materials pooled, contact angle and zeta potential of test organisms. | | | | | | | | | |
| --- | --- | --- | --- | --- | --- | --- | --- | --- | --- | --- |
| Organism | | | %R (SD  BB | %R (SD)  Artificial Test Soil^1^ | P^2^ | Contact angle (BB) | Contact angle (ATS) | Zeta potential (mV) BB | Zeta potential (mV) ATS | |
| KPC | | 0.5 (0.5) | | 6.9 (4.3) | <0.001 | 43.5 | 85.1 | -24.1 | | -18.4 |
| VRE | | 6.7 (4.7) | | 16.7 (9.1) | <0.001 | 28.3 | 51.3 | -30.3 | | -29.0 |
| AB | | 9.1 (3.3) | | 15.6 (5.6) | <0.001 | 53.3 | 75.0 | -25.9 | | -19.6 |
| CD | | 31.2 (10.0) | | 54.7 (10.9) | <0.001 | 79.6 | 78.3 | -4.6 | | -12.8 |

^1^represents ATS and ATS + dust data pooled

^2^ p= Mann-Whitney comparison of percent recovery of cells or spores deposited on given surface type when suspended in Butterfield’s Buffer (BB) and when suspended in Artificial Test Soil (Healthmark Industries, Inc. (ATS).
