# Supplementary material for: Factors influencing environmental sampling recovery of healthcare pathogens from non-porous surfaces with cellulose sponges
Source: PLoS One. 2022 Jan 13;17(1):e0261588. doi: 10.1371/journal.pone.0261588 (PMC8757884; doi:10.1371/journal.pone.0261588)
Supplement: S3 Table — (DOCX) [file pone.0261588.s003.docx]

S3 Table. Mann-Whitney comparison of percent recovery, as cultured on non-selective media, between surface types for each organism suspended in Artificial Test Soil (ATS) and spread evenly on surfaces.

| **C. difficile spores (CD)** | | | | |  | **A. baumanii (AB)** | | | | | | | |
| --- | --- | --- | --- | --- | --- | --- | --- | --- | --- | --- | --- | --- | --- |
|  | | | | |  |  | | | | | |  | |
|  | | p | |  |  |  | | | p | |  | |  |
| SS vs TP | | 0.021 | | TP higher %R |  | SS vs TP | 0.005 | | | SS higher %R | | | |
| SS vs WL | | 0.740 | | no difference |  | SS vs WL | 0.309 | | | no difference | | | |
| TP vs WL | | 0.006 | | TP higher %R |  | TP vs WL | 0.033 | | | WL higher %R | | | |
|  | | |  |  |  |  | | |  | |  | |  |
| TP highest, WL lowest | | |  |  |  | SS highest, TP lowest | | | | | |  | |
|  | | |  |  |  |  | | |  | |  | |  |
| **K. pneumoniae (KPC)** | | | | |  | **E. faecalis (VRE)** | | | | | | | |
|  | | | | |  |  | | | | | |  | |
|  | p | | |  |  |  | | | p | |  | |  |
| SS vs TP | 0.031 | | | SS higher %R |  | SS vs TP | | 0.018 | | SS higher %R | | | |
| SS vs WL | <0.001 | | | SS higher %R |  | SS vs WL | | 0.207 | | no difference | | | |
| TP vs WL | 0.001 | | | TP higher %R |  | TP vs WL | | <0.001 | | WL higher %R | | | |
|  | | |  |  |  |  | | |  | |  | |  |
| SS highest, WL lowest | | | |  |  | WL highest, TP lowest | | | | |  | |  |

SS: Stainless Steel, TP: Textured Plastic, WL: Wood Laminate
